# Comparative effects of music therapy and aromatherapy on stress, quality of life, and happiness among shift nurses in Korea: a randomized controlled trial

**DOI:** 10.7586/jkbns.24.030

**Published:** 2024-11-26

**Authors:** So-heun Lee, Won-jong Kim, Eun-Hi Choi, Myung-Haeng Hur

**Affiliations:** 1 Uijeongbu Eulji Medical Center, Eulji University, Uijeongbu, Korea; 2 Department of Nursing, Gimcheon University, Gimcheon, Korea; 3 College of Nursing, Eulji University, Uijeongbu, Korea

**Keywords:** Aromatherapy, Happiness, Music therapy, Quality of life, Stress, psychological

## Abstract

**Purpose:**

It is important to reduce stress and improve quality of life for shift nurses, who experience high levels of occupational stress. Identifying evidence-based interventions to enhance their well-being is crucial for both individual and healthcare system outcomes. This study aimed to evaluate the effects of music therapy and aromatherapy on stress, quality of life, and happiness among shift nurses.

**Methods:**

In this randomized controlled trial, participants were randomly assigned using computer-generated random numbers. A total of 78 shift nurses who met the inclusion criteria were enrolled. The experimental treatment involved providing music therapy for one week to the music group and aromatherapy for one week to the aroma group, whereas the control group followed their usual daily routines for one week.

**Results:**

No significant differences were observed among the three groups concerning general characteristics and baseline dependent variables, thereby confirming the homogeneity of the groups. Nevertheless, post-intervention analyses demonstrated significant improvements in perceived stress (F = 5.55, *p* = .006), stress index (F = 3.38, *p* = .039), quality of life (F = 11.79, *p* < .001), and happiness (F = 9.29, *p* < .001) in the experimental groups.

**Conclusion:**

Both music therapy and aromatherapy were found to be effective in mitigating stress and improving quality of life and happiness among shift nurses. Therefore, these interventions can be regarded as valuable strategies for improving overall well-being within this population.

## INTRODUCTION

Nurses function in a high-stress environment characterized by substantial physical, mental, and emotional demands, resulting in perceived fatigue levels significantly higher than those of workers in other professions [1]. Given that stress and fatigue contribute to various health issues among nurses and recognizing the correlation between nurses' health and patient outcomes, identifying effective methods for alleviating stress and fatigue is imperative [2].

Nurses working in hospitals care for patients with serious illnesses every day and interact directly with the families of patients at the end of life, which exposes them to various stressors [3]. Additionally, advances in medical technology have increased the number of tools that nurses must manage, and difficulties in communicating with healthcare professionals in different departments add to the everyday stress they experience [3]. Long-term exposure to these stressors can lead to physical health problems, such as stomach ulcers, heart disease, and high blood pressure, as well as mental health issues like depression, insomnia, and anxiety. This highlights the need for effective strategies to manage stress and promote overall well-being [4]. Shift nurses, in particular, experience irregular sleep patterns and sleep loss due to the nature of their work, which can result in sleep deprivation [5]. Additionally, high-intensity and continuous stress can activate the sympathetic nervous system, leading to poor sleep quality [5]. In response to sustained stress, the body manifests various physical symptoms such as headaches, insomnia, fatigue, gastrointestinal discomfort, and musculoskeletal pain as well as physiological responses such as increased heart rate, elevated blood pressure, and muscle tension through the autonomic nervous system and the hypothalamic-pituitary-adrenal axis [6]. When the homeostatic balance of the body is disrupted by heightened stress, reduced sleep quality, and fatigue, the overall quality of life (QoL) diminishes [5].

The QoL of the nurses in their job as healthcare providers is highly related to both nurses’ own comfort as well as patient comfort [7]. Shift work leads to irregular life patterns that can negatively affect nurses’ satisfaction with life and lower their QoL, which can also decrease their interest in patients and have negative effects on overall patient care [7,8]. Nurses with a high QoL are better capable of providing high-quality nursing services in response to patients’ requirements, which in turn enhances their satisfaction and further increases the productivity and efficiency of nursing care [8,9]. Accordingly, when nurses’ QoL is high, their happiness and satisfaction with life increase, leading to positive outcomes, such as job satisfaction, high-quality nursing care, and reduced turnover intentions; as such, QoL management is important [8,10].

Apart from having lower QoL, nurses also score lower on the happiness index than members of the general public, and nurses with low happiness scores present low job satisfaction and high turnover intentions [10]. Decreased happiness can lead to sleep disorders, anxiety, and depression; moreover, it can cause burnout at work, which has a detrimental effect on work performance [11]. Low happiness has also been reported to cause a decrease in nurses’ QoL, which adversely affects their productivity, patients, and organizations [12]. Happiness reduces stress, improves mental and physical health, and has positive impacts on life and job satisfaction for most people, including nurses [12]. Thus, interventions are needed that boost nurses’ happiness, reduce stress, improve their QoL, and encourage their efficiency during work [7,12].

Historically, psychiatric drug therapy has been the primary method for treating stress-related conditions and promoting mental health; however, its high costs and potential adverse effects have led to growing interest in complementary and alternative therapies [13].Thus, there is interest in integrative interventions that apply complementary and alternative therapies in the medical community and among the general public [13]. Complementary and alternative therapies include energy therapies, such as magnetic field therapy, acupuncture and meridian therapies; manual therapies, such as scent therapy and massage therapy; biological therapies that involve herbs and vitamins; and mind-body therapies, such as relaxation and meditation therapies [14,15].

Among such alternative therapies, music therapy, effective in reducing blood pressure and alleviating symptoms of depression, and aromatherapy, beneficial for stress and immunity management, are receiving increasing research attention [16-18]. Music therapy positively influences the brain and autonomic nervous system, effectively reducing anxiety, relieving stress, and improving symptoms of depression [19-21]. One advantage of music therapy is that participants can select and listen to music according to their mood or emotional state without limitations of time and place [22,23]. In aromatherapy, essential oils extracted from plants are utilized to promote mental and physical well-being [24]. Aromatherapy has been shown to relieve stress and negative emotions, improve sleep quality, and enhance immunity; through the commonly used inhalation method, individuals can easily apply therapeutic essential oils without any formal training [17,25].

While numerous prior studies have established the effectiveness of aromatherapy and music therapy as means of stress reduction [26,27], investigations into their impacts on happiness are relatively scarce. One notable study has compared the effectiveness of these two complementary and alternative therapies but only assessed the effectiveness of a one-time intervention [17]. Based on prior research suggesting that a week of music therapy or aromatherapy can enhance stress levels [28,29], this study applied both therapies over a one-week duration. To address these gaps in the literature, this study aims to provide foundational data on intervention methods that can effectively reduce stress and enhance happiness among shift nurses by examining the effects of music therapy and aromatherapy on the stress levels, QoL, and happiness of participating shift nurses.

## METHODS

### 1. Study design

**Figure 1.**
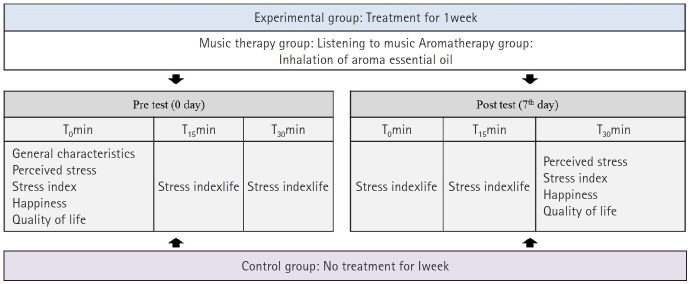
Research design. T_0_min = Before the experiment; T_15_min = 15 min after experimental treatment; T_30_min = 30 min after experimental treatment.

This randomized controlled trial (RCT) investigated the effects of music therapy and aromatherapy on stress, happiness, and QoL in shift nurses (Figure 1).

### 2. Participants

A total of 78 nurses with no hearing or olfactory impairments, who were working at Eulji University Hospital in Uijeongbu City, South Korea, voluntarily participated in the study. Nurses who were currently diagnosed with and receiving treatment for a medical illnesses, who were being treated with sleeping pills or anti-anxiety drugs, who consumed alcohol, or who were pregnant were excluded.

### 3. Sample size and randomization

**Figure 2.**
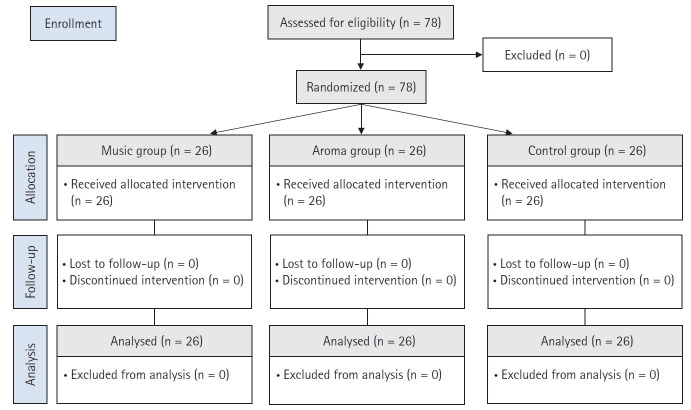
Process flow diagram.

The optimal sample size was calculated using G*Power 3.1.9.2 software, with the F-test and analysis of variance (ANOVA) (repeated measures) selected, an effect size set at .25, a two-tailed test power at .95, a significance level at .05, three groups (music therapy group, aromatherapy group, and control group), four variables and two measurements. Based on these parameters, a total of 66 participants were determined to be necessary, with 22 in each group. To account for a 20% dropout rate, 78 nurses were recruited, and none of them withdrew from the study; 26 nurses were randomly assigned to each group. Participating nurses were recruited by convenience sampling through announcements posted in the elevator and on the nursing ward bulletin boards at the hospital. After creating a list of participants in a protected Microsoft Excel 2021 document, random number generation was applied to randomly assign 26 people each to the music therapy, aromatherapy, and control groups. Pretest data collection was conducted with the control group first to prevent them being inadvertently exposed to the treatment (Figure 2).

### 4. Interventions

The study was conducted in a laboratory measuring 11.72 m^2^. To ensure a comfortable environment for participants, the room temperature was maintained between 22°C-24°C, and the indoor humidity was controlled at 40%-60%. All three groups were invited into the same room sequentially: first the control group, followed by the music therapy group, and finally the aromatherapy group, to prevent contamination and exposure to the aroma.

In the music therapy group, an initial selection of 15 songs was made based on consultations with a music expert, and these audio tracks were provided to the participants. Prior to the experiment, participants also selected an additional seven songs. The music therapy intervention was administered to this group three times daily for a duration of at least 30 minutes per session over the course of one week. This 30-minute listening period was established based on prior research that demonstrated a significant reduction in anxiety among surgical patients following a single 30-minute music listening session [30].

Aromatherapy is known to be most effective when two to three essential oils are blended [24]. Therefore, in consultation with an aromatherapist, an essential oil blend of lavender, ylang ylang, and lemon was prepared in a 5:5:1 ratio and stored in a light-protective container under refrigeration for use during the intervention. Participants in the aromatherapy group experienced continuous exposure to the aromatherapy through the use of an aromatherapy necklace, while the control group maintained their usual daily activities without any experimental intervention for the duration of the week.

The control group spent the week living their daily lives without experimental intervention (Table 1).

### 5. Instruments

#### 1) Primary outcome

##### (1) Stress

Both perceived stress and stress index were measured in the present study. perceived stress was measured using a stress scale developed by Park [31] that comprises a total of 30 items, including 15 items each on psychological stress and physical stress. Each item is scored on a 4-point Likert scale, from 1 “never” to 4 “always.” A higher score indicates a higher level of stress. The reliability of the scale (Cronbach’s ⍺) was .86 at the time of development and .93 in this study.

Stress index was measured by placing a pincer-type sensor into the finger using a Canopy9 RSA pulse rate monitor (Canopy9 RSA, IEMBIO Co., Chuncheon, Korea), a device designed to assess the autonomic nervous system. The stress index is a quantification of the ratio of sympathetic and parasympathetic nerve activity according to heart rate variability [32]. The stress index is calculated on a scale from 1~10, with higher values indicating higher levels of stress.

#### 2) Secondary outcomes

##### (1) QoL

In this study, the QoL of the nurses refers to their score on the Korean World Health Organization Quality of Life Brief (WHOQOL-BREF) version, which was developed by the WHO [33], and modified and supplemented by Min et al.[34]. The Korean version of the simplified QoL scale includes a total of 26 items in five domains: psychological health (six items), physical health (seven items), environment (eight items), social relations (three items), and overall QoL (two items). Each item is scored on a 5-point Likert scale, from 1 “strongly disagree” to 5 “strongly agree.” A higher score indicates a higher QoL. Items 3, 4, and 26 were reverse scored. Cronbach’s ⍺ at the time of development, excluding the items on overall QoL, was .76 in psychological health, .84 in physical health, .80 in environment, and .66 in social relations. Cronbach’s ⍺ was .90 in the study by Min et al.[34] and .92 in this study.

##### (2) Happiness

The Oxford Happiness Questionnaire, modified, supplemented, and adapted into Korean by Ahn [35] for nurses, consisting of 29 items, was used in this study. Each item is scored on a 4-point Likert scale, from 1 “strongly disagree” to 4 “strongly agree.” Higher scores indicate higher levels of happiness. Items 1, 5, 6, 10, 13, 14, 19, 23, 24, 27, 28, and 29 were reverse scored. Cronbach’s ⍺ was .91 in the study by Ahn [35] and .92 in the present study.

### 6. Data collection

Data were gathered between August 1 and 31, 2022. Perceived stress, stress index, QoL, and happiness were measured prior to the initiation of treatment and one week post- treatment. An environment was created to ensure that others would not be disturbed by being in the laboratory. The experimental treatments in this study included music therapy and aromatherapy for shift nurses according to the following procedures:

#### 1) Music therapy group

① The participant was guided to the laboratory and the experimental procedure was explained.

② Blood pressure was measured using an automatic electronic blood pressure monitor (BP170, Inbody, Cheonan, Korea) and stress index using a Canopy9 (Canopy9 RSA) autonomic nervous system measurement device.

③ The participant was provided with questionnaires and any guidance they needed to complete them to measure their perceived stress, QoL, and happiness.

④ The participant listened to music for 30 minutes using a personal mobile phone or wireless earphones (TEM-i7S, Shenzhen ITM Technology Co., Guangdong, China) as a music listening tool. The music comprised 15 songs primarily selected by the therapist-researcher from among the 32 songs in the “Isotonic Sound Series” created by Della Inc. and Sound Media, with the participant choosing an additional seven songs.

⑤ After 15 minutes of music listening, the stress index was measured using a Canopy9 device.

⑥ After 30 minutes of music listening, the stress index was measured using a Canopy9 device.

⑦ The participant was guided to listen to music at least three times a day for at least 30 minutes, including after work, during the one-week experimental period.

⑧ One week after the start of the experiment, blood pressure was measured using an automatic electronic blood pressure monitor and the stress index using a Canopy9.

⑨ One week after the experimental onset, the participant was provided with questionnaires and any guidance they needed to complete them to measure their perceived stress, QoL, and happiness.

⑩ The participant was informed that the experiment had concluded and was given coffee coupons as a reward. Aroma oil and a necklace were also made available to participants (excluding those in the aromatherapy group).

#### 2) Aromatherapy group

① The participant was guided to the laboratory and the experimental procedure was explained.

② Blood pressure was measured using an automatic electronic blood pressure monitor and the stress index using a Canopy9, an autonomic nervous system measurement device.

③ The participant was provided with questionnaires and any guidance they needed to complete them to measure their perceived stress, QoL, and happiness.

④ Three drops (0.3 cc) of a blended essential oil composed of lavender, ylang ylang, and lemon in a 5:5:1 ratio were applied to the aroma necklace. The participant was instructed to inhale the aroma for three minutes through the nose from a distance of 5–10 cm, and subsequently, to place it within 30 cm of the head to facilitate continuous inhalation.

⑤ After 15 min of aromatherapy application, the stress index was measured using a Canopy9 device.

⑥ After 30 min of aromatherapy application, the stress index was measured using a Canopy9 device.

⑦ The participant was guided to wear the aroma necklace at all times during the one-week experiment period, except during bedtime and bath time; it was explained that the aroma necklace should be kept upright while they were sleeping.

⑧ One week after the start of the experiment, blood pressure was measured using an automatic electronic blood pressure monitor, and the stress index was assessed using a Canopy9 device.

⑨ One week following the start of the experiment, the participant was provided with questionnaires and necessary guidance to complete them for measuring perceived stress, QoL, and happiness.

⑩ The conclusion of the experiment was explained to the participant, who was provided with coffee coupons as a reward.

### 7. Data analysis

Data collected in this study were analyzed using IBM SPSS Statistics version 26.0 (IBM Corp., Armonk, NY, USA). Participants' demographic characteristics were summarized using mean, standard deviation, frequency, and percentage. The homogeneity of the music therapy, aromatherapy, and control groups was assessed using the chi-square test (χ^2^-test) and one-way ANOVA. To evaluate the effectiveness of the experimental treatments, one-way ANOVA and repeated measures ANOVA were employed to analyze the stress, QoL, and happiness scores of the nurses across all three groups. Post hoc comparisons were conducted using the Scheffé test.

### 8. Ethical considerations

Before the trial commenced, the research protocol was submitted to and approved by the Institutional Review Board (IRB) of Eulji University (EU22-36). The study was subsequently registered with the Clinical Research Information Service (CRIS) (KCT0007944). Data collection for this study was conducted from August 1 to 31, 2022, within one year of the date of IRB approval. All nurses who responded to the recruitment notice posted in the hospital met the selection criteria and voluntarily consented to participate in the study. The purpose of the study, the procedures involved, and the possible adverse effects, such as low blood pressure or headache, were explained to the participants, and they were informed that they could withdraw from the study at any time. No adverse effects were reported during or after the experiment. The experiment was conducted after receiving consent forms from all the selected nurses who agreed to participate in the study. To protect participants’ privacy, a unique ID code was assigned to each participant, and the collected data were analyzed according to personal information processing guidelines and will be stored in a locked computer for three years before being discarded.

## RESULTS

### 1. Homogeneity test

A total of 78 nurses participated in this study, with 26 participants in each of the three groups: music therapy, aromatherapy, and control groups. Table 2 shows the general characteristics and dependent variables of the three groups.

Prior to treatment, the mean perceived stress score was 53.54 for the music therapy group, 57.62 for the aromatherapy group, and 53.65 for the control group, with no significant differences among the three groups. Prior to treatment, the mean stress index was 2.19 for the music therapy group, 2.38 for the aromatherapy group, and 1.85 for the control group, with no significant differences among the three groups. The mean QoL score was 90.88 for the music therapy group, 86.62 for the aromatherapy group, and 89.69 for the control group, with no significant differences among the three groups. The mean happiness score was 80.35 for the music therapy group, 75.65 for the aromatherapy group, and 81.73 for the control group, with no significant differences among the three groups.

The results of the homogeneity test on the participants’ general characteristics indicated no significant differences. The results of the homogeneity test on the dependent variables, perceived stress, stress index, QoL and happiness indicated no significant differences among the three groups, confirming homogeneity.

### 2. Effects of music therapy and aromatherapy on stress, QoL, and happiness

#### 1) Primary outcome

The mean perceived stress score after the experimental treatment was 42.46 for the music therapy group, 46.85 for the aromatherapy group, and 50.50 for the control group, indicating significant differences among the three groups (F = 3.71, *p* = .029). The results of multiple measurements of perceived stress during the seven days indicated significant differences according to time (F = 57.41, *p* < .001), and a significant interaction between group and time (F = 5.55, *p* = .006). The partial eta squared effect size of the experimental treatment according to group and time was .13. According to the post-hoc analysis results, the music therapy group had lower perceived stress after seven days of experimental treatment than the control group (*p* = .029) (Table 3).

The mean stress index after the experimental treatment was 2.42 for the music therapy group, 1.85 for the aromatherapy group, and 2.65 for the control group, indicating no significant difference among the three groups. According to the results of multiple measurements of participants’ stress index, while no significant difference was observed according to time, a significant interaction between group and time was found (F = 3.38, *p*= .039). The partial eta squared effect size of the experimental treatment according to time was .08 (Table 3).

#### 2) Secondary outcomes

The mean QoL scores after seven days of experimental treatment were 96.92, 91.62, and 85.50 for the music therapy, aromatherapy, and control groups, respectively, presenting significant differences among the three groups (F = 7.79, *p* < .001). Analysis of nurses’ pre- and post-intervention QoL scores revealed significant differences according to time, (F = 5.81, *p* = .018) and a significant interaction between group and time (F = 11.79, *p* < .001). The partial eta squared effect size of the experimental treatment according to group and time was .24. According to the post-hoc analysis, QoL was higher for the music therapy group after seven days of experimental treatment than for the control group, which received no treatment during the seven days of the experiment (*p* < .001) (Table 3).

The happiness scores after seven days of experimental treatment were 85.69, 78.69, and 79.46 for the music therapy, aromatherapy, and control groups, respectively; the scores differed significantly among groups (F = 3.18, *p* = .047). Analysis of pre-and post-intervention happiness scores indicated significant differences according to time, (F = 7.60, *p* = .007) and a significant interaction between group and time (F = 9.29, *p* < .001). The partial eta squared effect size of the experimental treatment according to group and time was .20 (Table 3, Appendix 1).

## DISCUSSION

After seven days of experimental treatment, perceived stress scores decreased by 11.08 for the music therapy group, 10.77 for the aromatherapy group, and 3.15 for the control group, compared with that before the experimental treatment. This is consistent with a previous study that reported decreases in perceived stress when intensive care unit nurses listened to their preferred music [22] and another study reporting decreased stress when nursing students inhaled lavender oil [29]. The music used in the present study has been found to promote the generation of alpha-waves, enhancing inner tranquility and peace through meditation, and can effectively contribute to mental and physical stability by applying various effective scientific principles, such as stereophonic sound [36]. Such results imply that music can stabilize the mind and body while positively reducing perceived stress, and that inhaling aroma oils enhances emotional relaxation and calm [37,38], making it effective in reducing perceived stress.

No significant differences were observed in stress Index among the three groups after seven days of experimental treatment. The results of this study are consistent with those of a previous study that reported no changes in the sympathetic and parasympathetic nervous systems after applying music therapy [39]; however, the current results differ from those of studies that reported a decrease in sympathetic nervous system activity and an increase in parasympathetic nerve system activity in the experimental group of healthy adults after music therapy [40]. The results of this study are consistent with studies showing that adults’ stress index decreased after aromatherapy treatment [22,29]. However, music therapy does seem to have various effects on the autonomic nervous system, with variables such as work intensity, personal environmental situation, and music preference affecting the autonomic nervous system. In addition, as the experimental environment in the current study differs from that in previous stress index studies, and none of them compared the stress of the participants who received music therapy and aromatherapy in the same environment, there is a limitation in interpreting these study results. Compared to previous studies using the same tool with nurses [5,41], the stress index was found to be low, while perceived stress levels were similar. The participants in this study are believed to be particularly sensitive to stress, as their perceived stress is high despite the low physiological stress index. In addition, in the stress index, there was no difference in the T0 of the pre-test and the T0 of the post-test in each group, so it can be interpreted that music therapy was only performed regularly and did not have a long-term effect. Future research should consider measuring baseline stress levels and conducting experiments with high-stress groups to assess the effectiveness of interventions. The lavender oil used in aromatherapy in this study is known to stimulate the parasympathetic nervous system [42] and have calming and relaxing effects on the central nervous system, as well as anti-depression and anti-anxiety effects [37]. The effects of ylang-ylang include emotional relaxation and the relief of nervous system tension [38], which seem to effectively reduce stress index.

These results indicate that music therapy is helpful in reducing perceived stress and that aromatherapy can be used as an effective intervention for relieving perceived stress as well as stress index. In particular, the results of this RCT indicated that stress was significantly reduced among the experimental groups compared with the control group. Thus, considering the intervention’s effects were verified to be long-lasting based on the present one-week study, long-term intervention strategies to lower subjective and objective stress are feasible.

According to the examination of the effects of music therapy and aromatherapy on the QoL of shift nurses, the QoL of the music therapy and aromatherapy groups improved after the experimental treatment, but there were no changes in the control group’s QoL. These results are similar to those of a study on music therapy for elderly people living alone [43] and an aromatherapy for pediatric with migraines [44].

QoL is a person’s perception of their overall condition [45]. The music used in this study was intended to generate A-waves [36] that improved nurses’ QoL by enhancing mental and physical stability and comfort. Based on evidence from previous studies related to individuals’ overall condition and QoL that various music-based therapies, such as singing and dancing therapies, are effective in improving QoL [43,46], it is expected that music programs that include singing, performance, and dancing are effective in improving QoL. Furthermore, among the essential oils used in the blend in this study, lavender and ylang ylang relieve stress, tension, headaches, and insomnia; affect the nervous system to alleviate emotions, such as depression, anxiety, and anger; and effectively evoke feelings of comfort [47,48], thereby improving sleep quality, and may effectively improve QoL by arousing happy emotions and moods. Thus, music therapy and aromatherapy are considered effective at increasing QoL. In this study, music appeared to provide emotional comfort and aromatherapy to provide relief from negative emotions.

As a result of the happiness scores, no significant differences were observed in the post hoc analysis; however, There was a significant difference between the three groups. The happiness scores of both the music therapy group and the aromatherapy group increased following the experimental treatment, while the happiness scores of the control group decreased. The results of this study are consistent with those of a study reporting that a song-centered music therapy program increased happiness and QoL among older adults [49]. The present findings are consistent with a previous study that found nurses’ happiness increased after inhaling aromatic oils [7]. Since there are few studies on the effects of music therapy on happiness, and most of those were conducted with infants or the older adults, the usefulness of comparing them with this study is limited. However, it has been reported that music-based activities induce positive emotions such as happiness and joy, and that happiness and satisfaction caused by these positive emotions have a substantial positive impact on QoL [49]. Thus, it is necessary to conduct research on the relationship between music therapy and happiness among young and middle-aged people in the future.

The music used in this study during the music therapy intervention has been reported to support mental and physical stability, reduce anxiety, and relieves tension and stress [36]. The essential oil blend used in the aromatherapy interventions has known positive effects as follows. Lavender relieves mental fatigue, anxiety and depression, and induces comfortable sleep, whereas ylang ylang relieves tension, provides a sense of comfort and happiness, and is effective for nervousness and insomnia [47,50], and lemon (used in smaller doses) can relieve the pain of migraines and headaches [48]. Thus, the nurses who used the music in this study may have experienced increased emotional stability and restoration, and relief from mental fatigue, and those who used the aromatherapy oils may have also experienced increased QoL and improved happiness through relief of physical and mental fatigue, thereby reducing insomnia and evoking a sense of happiness.

In conclusion, based on the present findings of increased QoL and happiness among nurses who received music therapy and those who received aromatherapy for one week, music therapy and aromatherapy seem to be effective in improving QoL and happiness for shift nurses. In addition, considering the scarcity of studies that used QoL and happiness as variables when examining the effects of music therapy and aromatherapy on population other than infants and older adults, the results of this study can serve as framework for the effects of music therapy and aromatherapy on QoL and happiness of younger adults.

This study is notable for comparing the effects of interventions using experimental music therapy and aromatherapy treatments on the interaction between time and group, and the findings verified the positive effects of prolonged (one-week) music therapy and aromatherapy. The study is also valuable because it incorporated both perceived stress and stress index as dependent variables.

The music used in the intervention is one of the study’s limitations. According to the experimental design, the researcher-therapist chose 15 songs initially, and then the research participants chose additional music; thus, their music preferences may have been overlooked. Future studies should consider including music preferences and varieties as study variables. Furthermore, the participants were recruited through convenience sampling and were limited to shift-nurses aged 23~32, restricting the generalizability of the findings. Another limitation of this study is that complete blinding was not achievable due to the nature of the experimental treatments.

## CONCLUSION

This study was an RCT that aimed to investigate the effects of music therapy and aromatherapy on stress, happiness, and QoL among shift nurses. When applied to shift nurses, music therapy and aromatherapy helped reduce perceived stress over time and improve QoL and happiness. As such, music therapy and aromatherapy are recommended for shift nurses to reduce their stress and improve their QoL and happiness.

## Figures and Tables

**Table 1.** Interventions for the Music Therapy Group, Aromatherapy Group, and Control Group

Variables	Music therapy group	Aromatherapy group	Control group
Laboratory environment	11.72 m^2^, temperature: 22~24°C, humidity: 40~60%		
Type of content	Isotonic sound series	Aroma essential oil lavender: ylang ylang: lemon	-
	(Created by Della Inc. and Sound Media)	= 5:5:1	
Tool	Personal mobile phone	Aroma necklace	-
	wireless earphones	(Three drops [0.3 cc] of a blended essential oil )	
Porcedures	(1) An initial selection of 15 songs was made based on consultations with a music expert	(1) The participant inhaled the aroma for 3 minutes through the nose from a distance of 5~10 cm	(1) The participant spent the week living their lives without experimetal invervention
	(2) The participant selected an additional 7 songs	(2) Place it within 30 cm of the head to facilitate continuous inhalation	
	(3) The participant listened to music for 30 minutes using a personal mobile phone or wireless earphones tool		
Dose	The participant was guided to listen to music at least 3 times a day for at least 30 minutes, including after work, during the one-week experimental period	The participant was guided to wear the aroma necklace at all times during the one-week experiment period, except during bedtime and bath time	-
Follow-up	Before the experiment		
	30 minutes after 1 week of the experiment		

**Table 2.** Homogeneity Tests of General Characteristics and Dependent Variables of Participants (N = 78)

Characteristics	Categories	Music therapy group (n = 26)	Aromatherapy group (n = 26)	Control group (n = 26)	F or* X^2^*	*p*
Sex	Men	5 (19.2)	4 (15.4)	5 (19.2)	0.17	> .999
	Women	21 (80.8)	22 (84.6)	21 (80.8)		
Age (yr)		24.00 ± 1.77	24.85 ± 2.20	24.46 ± 2.57	0.96	.387
Height (cm)		164.58 ± 6.61	162.69 ± 6.29	164.23 ± 7.44	0.57	.570
Body weight (kg)		60.12 ± 9.85	57.04 ± 10.15	59.92 ± 10.56	0.74	.479
Blood pressure (mmHg)	SBP	111.08 ± 11.21	110.73 ± 11.07	116.73 ± 11.60	2.31	.106
	DBP	74.04 ± 9.89	73.69 ± 12.14	77.15 ± 7.79	0.93	.400
Heart rate (bpm)		77.04 ± 11.94	77.69 ± 11.71	76.50 ± 7.32	0.08	.920
Perceived stress	D_0 _(T_0_)	53.54 ± 11.10	57.62 ± 13.68	53.65 ± 12.96	0.88	.420
Stress index	D_0 _(T_0_)	2.19 ± 1.23	2.38 ± 1.72	1.85 ± 1.08	1.03	.364
QoL	D_0 _(T_0_)	90.88 ± 10.88	86.62 ± 11.37	89.69 ± 11.96	1.01	.371
Happiness	D_0 _(T_0_)	80.35 ± 6.72	75.65 ± 12.63	81.73 ± 11.64	2.33	.105

Values are presented as the mean ± standard deviation or n (%). SBP = Systolic blood pressure; DBP = Diastolic blood pressure; bpm = Beats per minute; QoL = Quality of life; D_0_ = Before the experiment; T_0_ = Before the experiment.

**Table 3.** Comparison of Stress, Quality of life, and Happiness among the Three Groups (N = 78)

Variables		Music therapy group (n = 26)	Aromatherapy group (n = 26)	Control group (n = 26)	F (*p*)	F(*p*) ^‡^
Perceived stress	D_0_ (T_0_)	53.54 ± 11.10	57.62 ± 13.68	53.65 ± 12.96	0.88 (.420)	Time 57.41 (< .001)
						Group 1.33 (.272)
	D_7_ (T_30_)	42.46 ± 6.38^a†^	46.85 ± 12.82	50.50 ± 11.65^b†^	3.71 (.029)	G*T 5.55 (.006)
Stress index	D_0_ (T_0_)	2.19 ± 1.23	2.38 ± 1.72	1.85 ± 1.08	1.03 (.364)	Time 0.62 (.434)
						Group 0.27 (.766)
	D_7_ (T_30_)	2.42 ± 1.24	1.85 ± 0.93	2.65 ± 1.70	2.56 (.084)	G*T 3.38 (.039)
Quality of life	D_0_ (T_0_)	90.88 ± 10.88	86.62 ± 11.37	89.69 ± 11.96	1.01 (.371)	Time 5.81 (.018)
						Group 2.82 (.066)
	D_7_ (T_30_)	96.92 ± 9.80^b†^	91.62 ± 11.54	85.50 ± 9.89^a†^	7.79 (< .001)	G*T 11.79 (< .001)
Happiness	D_0_ (T_0_)	80.35 ± 6.72	75.65 ± 12.63	81.73 ± 11.64	2.33 (.105)	Time 7.60 (.007)
						Group 2.11 (.128)
	D_7_ (T_30_)	85.69 ± 8.13	78.69 ± 12.92	79.46 ± 11.33	3.18 (.047)	G*T 9.29 (< .001)

Values are presented as the mean ± standard deviation. D_0_ = Before the experiment; D_7_ = 7^th^ day of the experiment; T_0_ = Before the experiment; T_30_ = 30 minutes after the experiment; G*T = Group*Time. ^†^Means for each group with different superscripts (a,b) indicate a significant difference (Scheffé : *p* < .05). ^‡^Repeated-measures ANOVA.

## Data Availability

The data that support the findings of this study are available from the corresponding author upon reasonable request.
