# Supplementary material for: Comparative effects of music therapy and aromatherapy on stress, quality of life, and happiness among shift nurses in Korea: a randomized controlled trial
Source: J Korean Biol Nurs Sci. 2024 Nov 26;26(4):337–49. doi: 10.7586/jkbns.24.030 (PMC13268317; doi:10.7586/jkbns.24.030)
Supplement: Supplementary file 1 [file jkbns-24-030-Appendix-1.pdf]

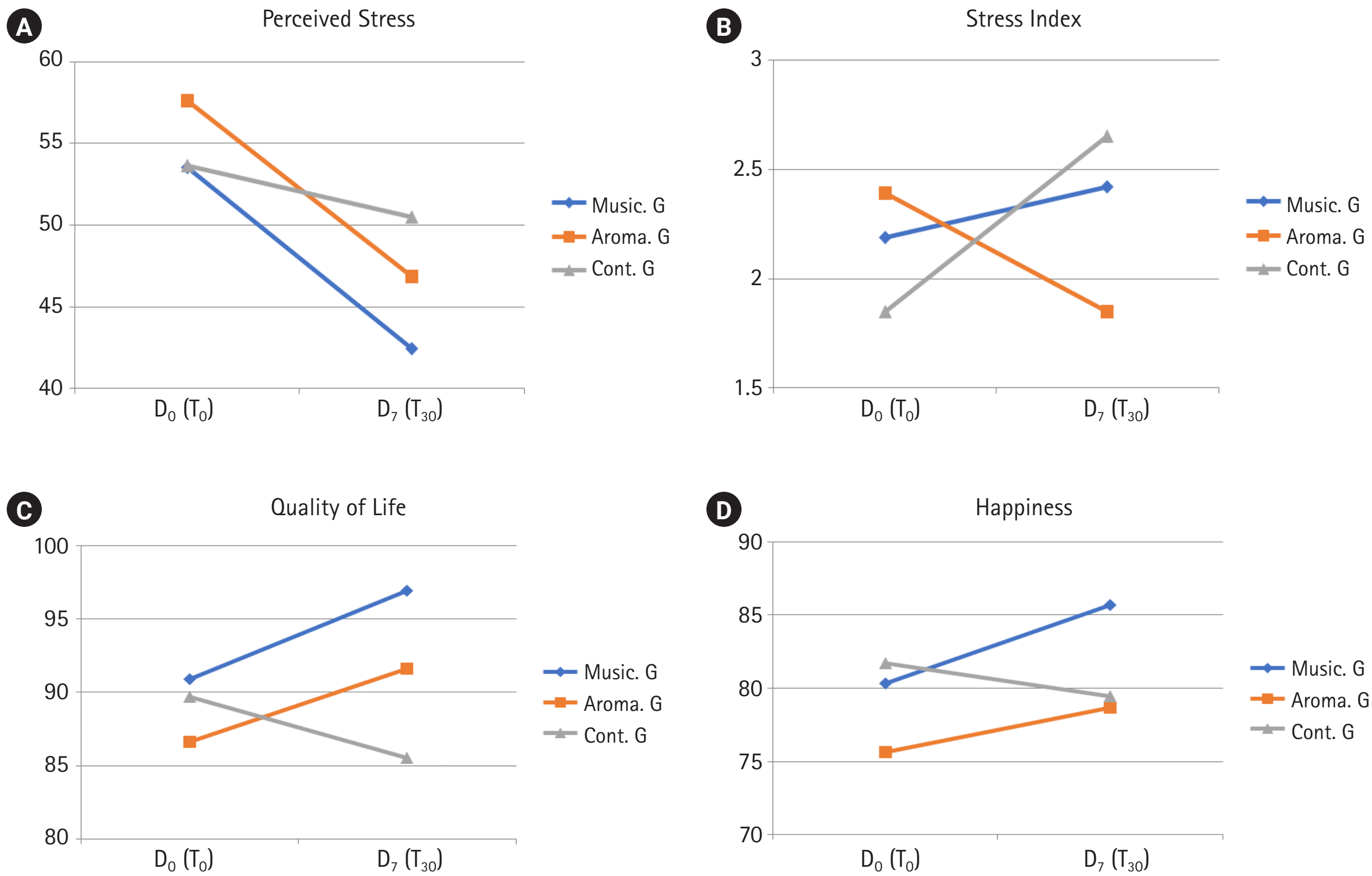

Appendix 1. Comparative effects of music therapy and aromatherapy on stress, quality of life, and happiness among three groups. (A) Perceived stress, (B) Stress index, (C) Quality of life, (D) Happiness.
Music G = Music group; Aroma G = Aroma group; Cont. G = Control group; D_0 = Before the experiment; D_7 = Experimental 7th day; T_0 = Before the experiment; T_{30} = 30 minutes after the experiment.
